# Integrated approach to hydrogeochemical appraisal of groundwater quality concerning arsenic contamination and its suitability analysis for drinking purposes using water quality index

**DOI:** 10.1038/s41598-023-40105-9

**Published:** 2023-11-22

**Authors:** Zahid Ullah, Xian-Chun Zeng, Abdur Rashid, Junaid Ghani, Asmat Ali, Muddaser Shah, Rimsha Zainab, Mikhlid H. Almutairi, Amany A. Sayed, Lotfi Aleya

**Affiliations:** 1 State Key Laboratory of Biogeology and Environmental Geology, School of Environmental Studies, China University of Geosciences, Wuhan, 430074 China; 2 Department of Botany, Abdul Wali Khan University, Mardan, 23200 Pakistan; 3 Natural and Medical Sciences Research Center, University of Nizwa, Birkat Al Mauz, P.O. Box 33, 616 Nizwa, Oman; 4 Department of Botany, Women University Swabi, Swabi, Khyber Pakhtunkhwa Pakistan; 5 Department of Zoology, College of Science, King Saud University, P.O. Box 2455, 11451 Riyadh, Saudi Arabia; 6 Zoology Department, Faculty of Science, Cairo University, Giza, 12613 Egypt; 7 Chrono-Environnement Laboratory, UMR CNRS 6249, Bourgogne, Franche-Comté University, CEDEX, 25030 Besancon, France

**Keywords:** Environmental sciences, Hydrology

## Abstract

Arsenic (As), contamination in drinking groundwater resources is commonly environmental problem in many developing countries including Pakistan, with significant human health risk reports. In order to examine the groundwater quality concerning As contamination, its geochemical behavior along with physicochemical parameters, 42 samples were collected from community tube wells from District Bahawalpur, Punjab, Pakistan. The results showed the concentration of elevated As, its source of mobilization, and associated public health risk. The As concentration detected in groundwater samples varied from 0.12 to 104 µg/L with an average value of 34.7 µg/L. Among 42 groundwater samples, 27 samples were beyond the permitted limit of 10 µg/L recommended by World Health Organization (WHO), for drinking purposes. Statistical analysis result show that the groundwater cations values are in decreasing order such as: Na^+^ > Mg^2+^ > Ca^2+^ > K^+^, while anions were HCO_3_^–^ > SO_4_^2–^ > Cl^–^ > NO_3_^–^. Hydrochemical facies result depict that the groundwater samples of the study area, 14 samples belong to CaHCO_3_ type, 5 samples belong to NaCl type, 20 samples belong to Mixed CaMgCl type, and 3 samples belong to CaCl_2_ type. It can be accredited due to weathering and recharge mechanism, evaporation processes, and reverse ion exchange. Gibbs diagram shows that rock water interaction controls the hydrochemistry of groundwater resources of the study area. Saturation Index (SI) result indicated the saturation of calcite, dolomite, gypsum, geothite, and hematite mineral due their positive SI values. The principal component analysis (PCA) results possess a total variability of 80.69% signifying the anthropogenic and geogenic source of contamination. The results of the exposure-health-risk-assessment method for measuring As reveal significant potential non-carcinogenic risk (HQ), exceeding the threshold level of (> 1) for children in the study area. Water quality assessment results shows that 24 samples were not suitable for drinking purposes.

## Introduction

Medical geology is a growing interdisciplinary scientific field exploring the influence of natural geological aspects and their effects on plant, animal, and human health. It deals with the impact of environmental factors on geographical distribution of health problems mainly in humans, which is a complex issue that requires broad approaches to solve the problems^1^. Medical geology is a comparatively advanced area of research in Pakistan, mostly in terms of assessing groundwater quality and its suitability for domestic and agricultural use, which requires a holistic approach from researchers^2^. Arsenic (As) is on special focus to reduce As related environmental, agricultural, and public health issues both worldwide and nationally^3^.

As and several As compounds have been recognized as Class-I human carcinogens by the International Agency for Research on Cancer (IARC)^4^. Considering the high carcinogenic risk of As’s, the World Health Organization (WHO) reduced the threshold value for As in drinking water to 10 µg/L in 1993, replacing the previous level of 50 µg/L^5^. Furthermore, on June 22, 2000, the US Environmental Protection Agency (New Jersey) also suggested a safe As levels of 5 µg/L in drinking water to properly protect public health^6^. Although geologically released As is a major source of groundwater pollution, several anthropogenic activities are also assumed to be responsible for As contamination in ground and surface water^7^. It is found in over 200 mineral forms in the geothermal system, but the most prevalent minerals are arsenical pyrite (FeAsS), orpiment (As_2_S_3_), and realgar (AsS)^8^. As release in aquifers could be triggered by geochemical changes in subsurface sediments, such as rock water interaction, sorption/desorption, and oxidative/reductive dissolving processes of As-bearing (FeAsS) and (Fe) oxides^9^.

High levels of As in groundwater is triggered due to oxidative desorption along with an increase in the hydrologic concentration process, as well as other physicochemical factors^10^. As exposure can result in skin lesions, pigmentation, hardening of the palms, and soles of the hands and feet, a condition known as hyperkeratosis, as well as multiorgan cancer^11^. Humans can be exposed to As through different pathways, including ingestion, inhalation, and skin contact^12^. Drinking As-contaminated water is one of the most serious risk to human health, as evidenced by a vast number of documented cases around the world and several national studies^13^. Medical geology using human health risk assessment numerical method to calculate the health risk exposure due to toxic elements, such as As which may cause various diseases in human health^14^.

Groundwater is a vital freshwater resource in Pakistan, it is strategically important due to its increasing demand in agriculture, residential, and industrial purposes. Pakistan, is in an arid climate zone, where average rainfall is less than 200 mm per year and groundwater availability is restricted^15^. Pakistan’s primary groundwater resources are irrigated areas of the Indus Basin, followed by places outside the basin. Peoples in some drought-affected areas of Pakistan, already lack fresh drinking water and must rely on saline water. As per estimates, 60% of Pakistan’s fresh water is destroyed both in terms of quality and quantity because of poor management, with just 40% used for home and industrial purposes. According to the Bureau of National Statistics, around 56% of the total population has access to safe drinking water^16^. However, according to international criteria, only 25.61% of the population has access to safe drinking water. From 2002 to 2006, the Pakistan Council of Research in Water Resources, (PCRWR), started a project to conduct a detailed investigation of the water quality in 23 major cities across the country, which was expanded to 25 cities in 2015 and beyond. According to the report, an average of 84–89% of the water resources in the country have water quality that falls below the required levels for human consumption^17^.

Pakistan, is also facing the problem in terms of As contamination in groundwater resources. In recent decades the groundwater resources of Pakistan, especially in Punjab Province, is significantly vulnerable due to rapid industrialization, mining activities, agricultural practices, which had made the groundwater resources vulnerable both in terms of quality and quantity. As contamination in groundwater resources is reported in many districts of Punjab, Pakistan, such as Multan^18^, Lahore^19^, Sialkot^20^, Jhelum^21^, Mandi Bahauddin^22^, Gujrat^14^, Sheikhupura^23^, but the study is still limited to investigate the realistic situation of groundwater quality of Punjab, Pakistan. It seems compulsory to conduct a detailed survey of groundwater resources concerning As contamination. Therefore, we choose District Bahawalpur, which is the 11th largest city in Punjab, Pakistan, with a population of 10 million in which 76% population rely on groundwater for domestic and ingestion motive^24^.

Keeping in view the situation of groundwater contamination this study aimed: (i) to investigate the concentration profile of As along with other physicochemical parameters in the groundwater samples of the study area, (ii) to investigate the source of mobilization of As in to the groundwater resources of the study area, (iii) to monitor the health risk index of As on the population of the study area, (iv) to identify the mineral phases of groundwater samples, and (v) to evaluate the suitability of groundwater for drinking purposes.

## Study area

### Location and climate

**Figure 1:**
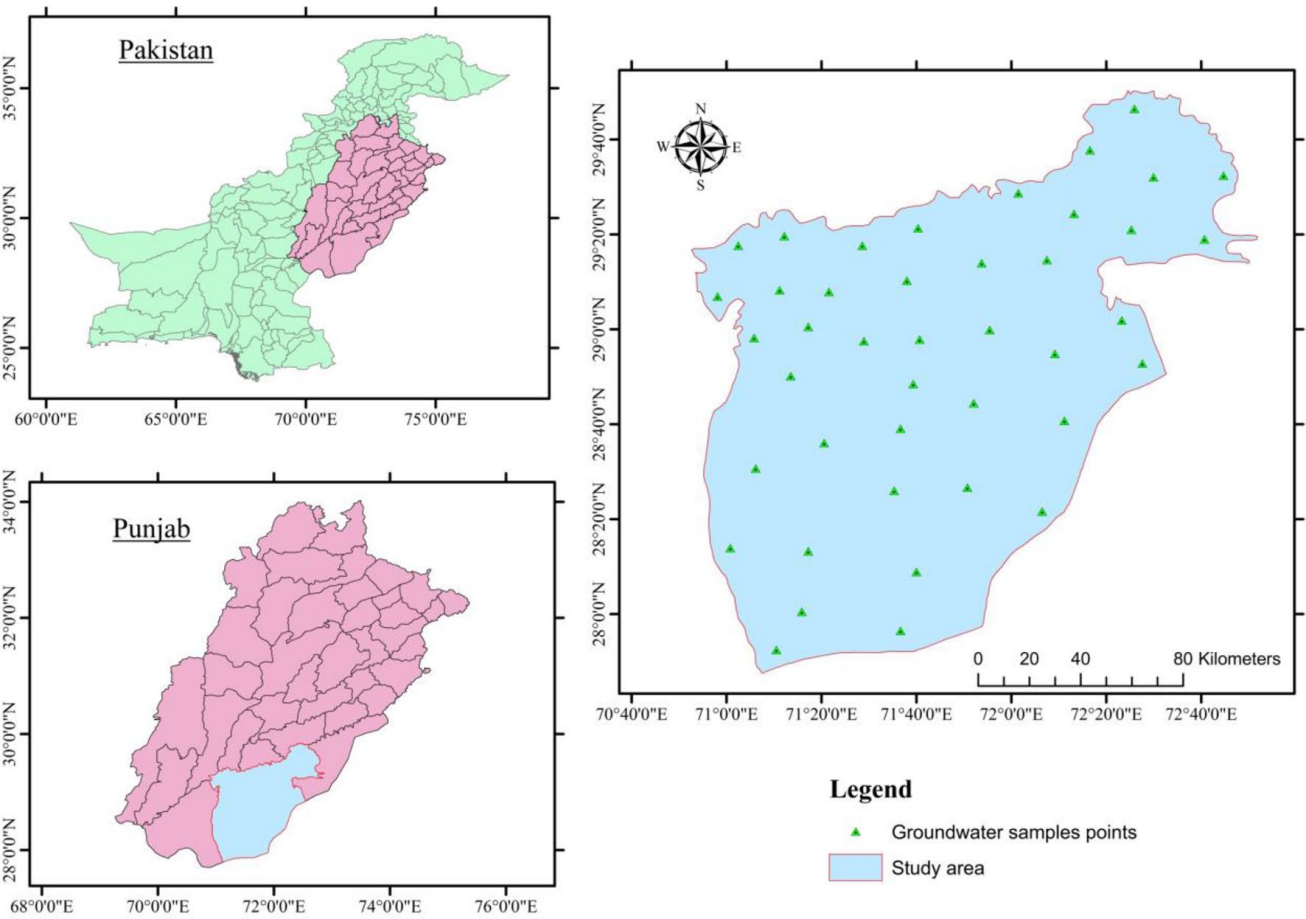
Geographical location of the study area along with groundwater sampling stations.

The current research was conducted in District Bahawalpur, which is located in Punjab’s southern region. It is located between the latitudes of 27°–80′ and 29°–50′ north and the longitudes of 70°–54′ and 72°–50′ east as shown in (Fig. 1). The climate in the region is hot and humid, with hot and rainy summers and dry, chilly winters with little precipitation. In the studied area, the annual average temperature and precipitation were 29.8°C and 410 mm, respectively. Wheat, gram, barley, rice, and sugarcane are the principal crops grown in the area.

### Geological settings of the study area

The study area is the part of upper Indus Plain, which is covered by alluvial strata. The ground slopes southwest at a rate of 0.3 to 0.4 m per kilometer. The nearby flooding plains rise to 50 feet in the center of the plain via terraces or bars. Exploratory drills revealed that a 1500-m-thick quaternary alluvium with scattered bedrock hills (Kharana Hills) had overlain Precambrian basement rocks, breaking the plain’s low relief. The area is covered in meander-belt deposits, stream-belt deposits, and flood-plain deposits. The soils are made up of alluvial material brought by the Indus River and its tributaries. Individual strata's vertical and horizontal continuity is limited in diverse alluvial deposits. The soil is primarily reddish-brown to grayish-brown in color, medium-textured, and contains a lot of fine to very fine sand, as well as tiny amounts of clay and gravel. Fine to medium sand, silt, and clay make up the majority of the alluvial complex. Silty or clayey sand is used to incorporate siltstone and mudstone pebbles. Despite their diversity, the alluvial deposits constitute a coherent unconfined aquifer that is very transmissive. The strata above 91 m are compacted but extremely productive. The water table in the area varies in depth from 2 to 7 m, with an average of 3.5 m.

### Formation and lithology

The huge Indus River, alluvial plain comprises an area of around 100,000 square miles and is composed of fluvial deposits that range in thickness from 590 m to more recent times. The Tethys Sea, which formerly reached as far as Pakistan’s northern border but gradually withdrew as the Himalayas expanded, formed the area. The majority of the rocks are marine, severely bent, fragile, and shattered all over^25^. They are primarily made of clay and limestone. According to lithological research, the subsurface contains a sandy layer that is 200–400 feet thick. They are fine to medium micaceous sands with lenses and bands of silt and silty clay that have been well separated^26^. It is challenging to link the strata found in two nearby boreholes due to the high variability of the deposits and the lithology. Over a broad area, the proportion of sand and clay bands is extremely stable. In this aquifer, the depth to the water table varies from 1.56 m to 11.93 m, with an average depth of 4.53 m^27^.

### Land use

**Figure 2:**
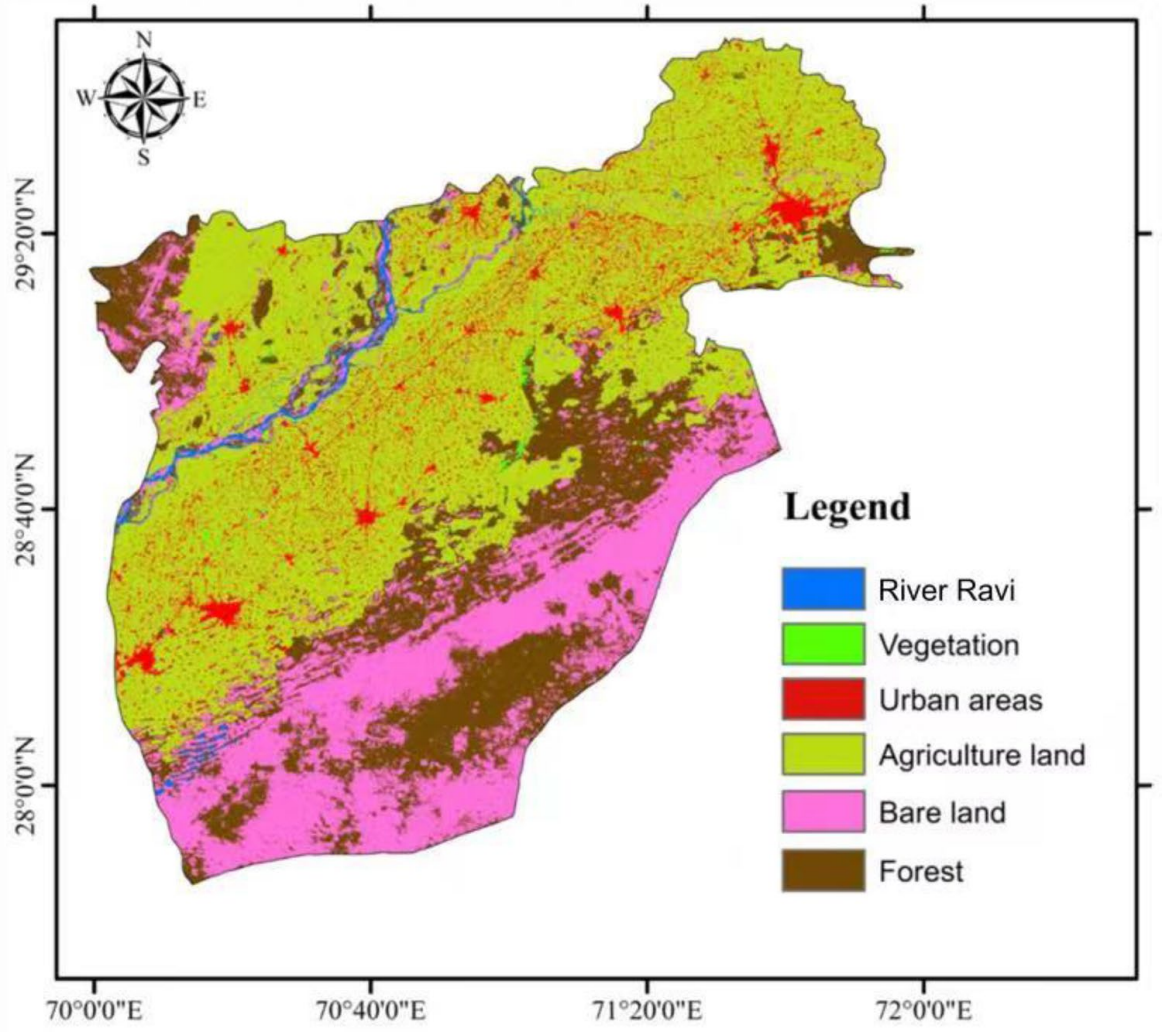
Land use map of the study area.

The supply of an ecosystem’s services is affected when land use patterns and processes are changed. Climate change has an impact on ecosystem services either directly or indirectly through changes in greenhouse gas concentrations and hydrological processes^28^. The classification of land used of the study area is shown in (Fig. 2). In the study area the alluvial plains have been changed into cultivated lands. The most common crops of the study area are vegetables, sugarcane, wheat, and rice. The unfertile land in the study area has been seen in the form of eroded land with sand and silt. River Ravi, and its tributaries are the water channels in the study area^29^. The industrial area contains various industries like textile, shoes, food processing industries. In the study area there are many factors that contribute to land use change, such as urbanization, soil and water erosion, tree cutting, overgrazing, poultry discharge into streams, fragmentation of aquatic habitats, water pollution caused by deforestation, discharge of untreated municipal and industrial wastewater, and pesticide residues^30^.

## Material and methods

### Sample collection and analysis

The current research work was conducted in District Bahawalpur, of Punjab, Pakistan. A total of (n = 42) samples were collected from community tube wells to access the groundwater quality of the study area. The groundwater samples were collected in polyethylene contamination free bottles having a capacity of 1.5 L. Prior to sampling the wells, were started for 5–10 min flow to avoid the effect of stagnant water^31^. The samples were initially filtered using a 0.45 µm membrane and then kept in ice-box. All the physicochemical parameters were measured using standard reference material as shown in Table S1. The basic water quality parameters like pH, total dissolved solids (TDS), and electrical conductivity (EC) were examined using multi-parameter analyzer (Hanna HI9829), following^32^. The groundwater samples were shifted immediately to a standard water quality laboratory Pakistan Council of Research on Water Resources (PCRWR), to measure the concentration of major cations, anions and (As). An ultraviolet spectrophotometer was used to measure nitrate (NO_3_^–^) and sulfate (SO_4_^2–^) levels. Titration method was used to determine the concentrations of chloride (Cl^–^) and bicarbonate (HCO_3_^–^). A volumetric titration with ethylene diamine tetra-acetic acid (EDTA, 0.05 N) was used to assess important cations such Mg^2+^ and Ca^2+^, with a ≤ 2 percent analytical error^33^. A flame photometer was used to measure Na^+^ and K^+^ levels. An atomic absorption spectrophotometer (AAS Vario 6 Analytik Jena AG), a mercury hydride setup HS55, and 99.9% pure argon (Ar) gas were used to detect arsenic content^34^. The apparatus was calibrated with known multiples before analysis using standard stock solutions that have been certified. Equation (1) was used to compute the charge balance error for each sample.

1 $${\text{CBE }} = \, \left( {\sum {\text{cations}} - \sum {\text{anions}}} \right)/\left( {\sum {\text{cations}} + \sum {\text{anions}}} \right) \, \times {1}00,$$

All ionic concentrations are expressed in milliequivalents per liter (meq/L). For further investigation, samples (n = 42) within ± 5 CBE ranges were chosen.

### Quality assurance and quality control

Routine quality control checks, standardized operating protocols, reagent blanks, standard calibration, and triple analyses were used to achieve accuracy and precision in the results of analytical data following^3^. The chemicals used in the analysis were bought from Germany (Merck Company). To remove the contaminants, all glassware was thoroughly washed with deionized water and a 30 percent HCl solution. Glassware was oven-dried after being washed. After six samples, reagent blanks were used to monitor and evaluate contamination, and the concentration of the blank was subtracted from the groundwater concentration^8^.

### Statistical and hydrogeochemical analysis

Statistical analysis plays a vital role in interpretation of data set by representing various acts^35^.

The SPSS software version (v23, SPSS Inc., Armonk, NY, USA) was used to conduct regression, Principal component analysis and Pearson correlation analysis to identify the pollution sources, connections between water quality parameters. Aqua-Chem (version 2010.1, Waterloo Hydrogeologic, Kitchner, Ontario, Canada) was used to create the Piper diagram for hydrochemical facies interpretation^36^. The geochemical modeling program PHREEQC (version 3.1) was used to calculate saturation indices, which indicate the tendency of groundwater to dissolve or precipitate a particular mineral^37^. Gibbs^38^ diagram was prepared over Grapher (version 14), to understand the controlling mechanism of groundwater chemistry. The study area map and land use map were created over ArcGIS software (version 10.8, USA).

### Human health risk assessment

To document the toxicity level of As in groundwater, a health risk assessment was conducted. Humans are exposed through three different routes such as drinking, skin contact and inhalation. Oral intake is still the most vulnerable among these three routes. In this study we calculate the human health risk of As via oral pathway in terms of chronic daily intake (CDI), hazard quotient (HQ), and cancer risk (CR) using following equations.

2 $$CDI=\frac{C\times IR}{BW},$$

3 $$HQ=\frac{CDI}{RfD} ,$$

4 $$CR=CDI\times CSf,$$

where *C* is the As concentration (µg/L) in groundwater, *BW* is body weight (70 kg) for adults and (32.7 kg) for children, *IR* is the ingestion rate (2 L day^–1^), *CSF* refers to cancer slope factor (1500 (µg kg day^–1^)^–1^, and *RfD* is the oral reference dose (0.3 µg kg day^–1^)^39^.

### Water quality index (WQI)

The water quality index (WQI) values were used to determine the appropriateness of groundwater for drinking. The WQI was calculated using the WHO drinking water standard from (2011)^40^. Equation (5) was used to get the WQI values.

5 $$\mathrm{WQI}=\sum_{\mathrm{i}=1}^{\mathrm{n}}{\mathrm{SI}}_{\mathrm{i}} .$$

## Results and discussion

### Geochemical composition of groundwater

Table 1 shows the concentrations of selected physicochemical parameters in the groundwater samples collected from the study area. The pH value in the groundwater samples ranges between 7.36 and 8.22 with mean value of 7.71. The value of pH in all groundwater samples were inside the permissible limit recommended by world health organization (WHO)^41^. As a key water quality parameter pH determination is compulsory due to its vital role in the saturation of groundwater physicochemical parameters^23^. The value of EC varied between 518 and 2820 mS/m with an average value of 1141 mS/m, and were recorded beyond the permitted limit recommended by (WHO)^41^. The elevated concentration of EC in groundwater is due to saline condition of the groundwater resources^31^. Turbidity values ranges between 0.09 and 7.74 with an average value of 1.82 Nephelometric Turbidity Units (NTUs). Surface recharge, water runoff, weathering processes, and industrial effluents are all contributing to the high turbidity in the groundwater resources of the study area. Furthermore, high levels of turbidity is caused due to poorly designed wells^42^. The concentration of TDS in groundwater samples varied between 332 and 1805 mg/L, with an average value of 730 mg/L, most of the samples were inside the WHO permitted limit^41^. The elevated concentration of TDS in groundwater is due to ion dissolution which might be attributed to the gradual depletion of salts and minerals over time^39^. The depth of tube-wells in the study area varied from 37 to 80 (m) with an average value of 39 (m). The concentrations of Na^+^, K^+^, Cl^–^ and HCO_3_^–^ in groundwater samples varied from 27 to 483 mg/L, 1.3–13.4 mg/L, 13–134 mg/L, and 160–560 mg/L, respectively, with an average value of 122 mg/L, 6.58 mg/L, 46.9 mg/L and 301 mg/L respectively. Except HCO_3_^–^ the concentrations of Na^+^, K^+^, and Cl^–^ were inside the acceptable limit recommended by WHO^41^. However, the dissolution of calcite, carbonate, marble, and dolomite-bearing minerals causes an increase in HCO_3_^–^ concentration^18^. The concentrations of SO_4_^2-^, Mg^2+^, Ca^2+^, and NO_3_^–^ in groundwater samples ranges between 13–370 mg/L, 12–112 mg/L, 24–100 mg/L, and 0.10–0.40 mg/L respectively, with mean values of 105 mg/L, 44.1 mg/L, 52.2 mg/L, and 0.18 mg/L, respectively. The concentrations of SO_4_^2–^, Mg^2+^, Ca^2+^, and NO_3_^–^ in groundwater samples were inside the acceptable limit recommended by WHO. The concentration of Fe^2+^ in groundwater samples varied between 0.03 and 4.75 mg/L, with mean value of 0.60 mg/L, and was beyond the permitted limit of WHO, for drinking purposes. The influencing factor of Fe^2+^ in groundwater resources are natural deposits, iron-bearing industrial wastes, dissolving effluents, and acidic mine drainage^43^. Fluoride (F^-^) concentration in groundwater samples ranges between 0.15 and 6.05 mg/L, with an average value of 0.76 mg/L, and reported inside the permissible limit of 1.5 mg/L recommended by (WHO)^41^. However, elevated concentration of F^–^ in groundwater is due to fluorite bearing minerals, rock-water interaction and ion exchange processes^44^. Among cations, Na^+^ shows a higher mean concentration 122 mg/L, followed by Ca^2+^ 52.2 mg/L, Mg^2+^ 44.1 mg/L, and K^+^ 6.58 mg/L in all groundwater resources and was within the recommended acceptable range of (WHO), while in anions HCO_3_^–^ shows a higher concentration with an average value of 301 mg/L, followed by SO_4_^2–^ 105 mg/L, Cl^–^ 46.9 mg/L, NO_3_^–^ 0.18 mg/L, all the major anions were in WHO permitted limit except HCO_3_^–^.

**Table 1:** Descriptive statistics of selected parameters in groundwater samples collected from the study area.

Parameters	Minimum	Maximum	Median	Mean	SD	WHO
pH	7.36	8.22	7.66	7.71	0.24	6.5–8.5
EC µs/cm	518	2820	955	1141	623	400
Turbidity	0.09	7.74	1.19	1.82	2.03	5.00
TDS (mg/L)	332	1805	611	730	398	1000
Depth (m)	37	80	35	39	18.6	–
Na^+^ (mg/L)	27	483	93	122	101	200
K^+^ (mg/L)	1.3	13.4	6.1	6.58	3.68	12.0
Cl^-^ (mg/L)	13	134	38	46.9	31.3	250
HCO_3_^–^ (mg/L)	160	560	270	301	101	250
SO_4_^2–^ (mg/L)	13	370	91	105	79.1	250
Mg^2+^ (mg/L)	12	112	39	44.1	30.0	50
Ca^2+^ (mg/L)	24	100	48	52.2	19.0	100
NO_3_^–^ (mg/L)	0.10	0.40	0.2	0.18	0.08	10
Fe^2+^ (mg/L)	0.03	4.75	0.2	0.60	1.02	0.30
F^–^ (mg/L)	0.15	6.05	0.46	0.76	1.14	1.50
As (µg/L)	0.12	104	35	34.7	30.3	0.10

### Arsenic contamination in groundwater

Arsenic contamination in potable groundwater resources could make unfit for drinking purposes and may cause various health diseases, like kidney failure, heart problems, and hair loss^7^. In the present study As contamination in the community tube-wells of the study area varied between 0.12 and 104 µg/L, with mean value of 34.7 µg/L, 27 samples were beyond the recommended value of WHO for drinking purposes. Elevated concentration in drinking groundwater resources is a direct consequence of geogenic and anthropogenic sources in the study area. As release in groundwater may also occur in high salinity, alkalinity, and anoxic settings^14^. Geogenic arsenic pollution of groundwater is more widespread in alluvial aquifers. These aquifers are largely gravel, sandstone, silt, and sand that have been trapped in a river channel or flood plain for an extensive period^19^. The elevated levels of HCO_3_^–^ in groundwater under highly alkaline conditions could be associated with precipitation of calcite and dolomite, resulting in adsorption of As on calcite. The availability of microorganisms improves the reductive decomposition of pH-based iron hydroxide and the absorption of bicarbonate minerals. Similarly, natural arsenic mobilization in the groundwater is aided by evaporation and rock-water interaction^3^. Anthropogenic source of As contamination in groundwater sources are mining actions, industrial effluents, and agricultural pesticide’s^31^.

### Hydrochemical facies

**Figure 3:**
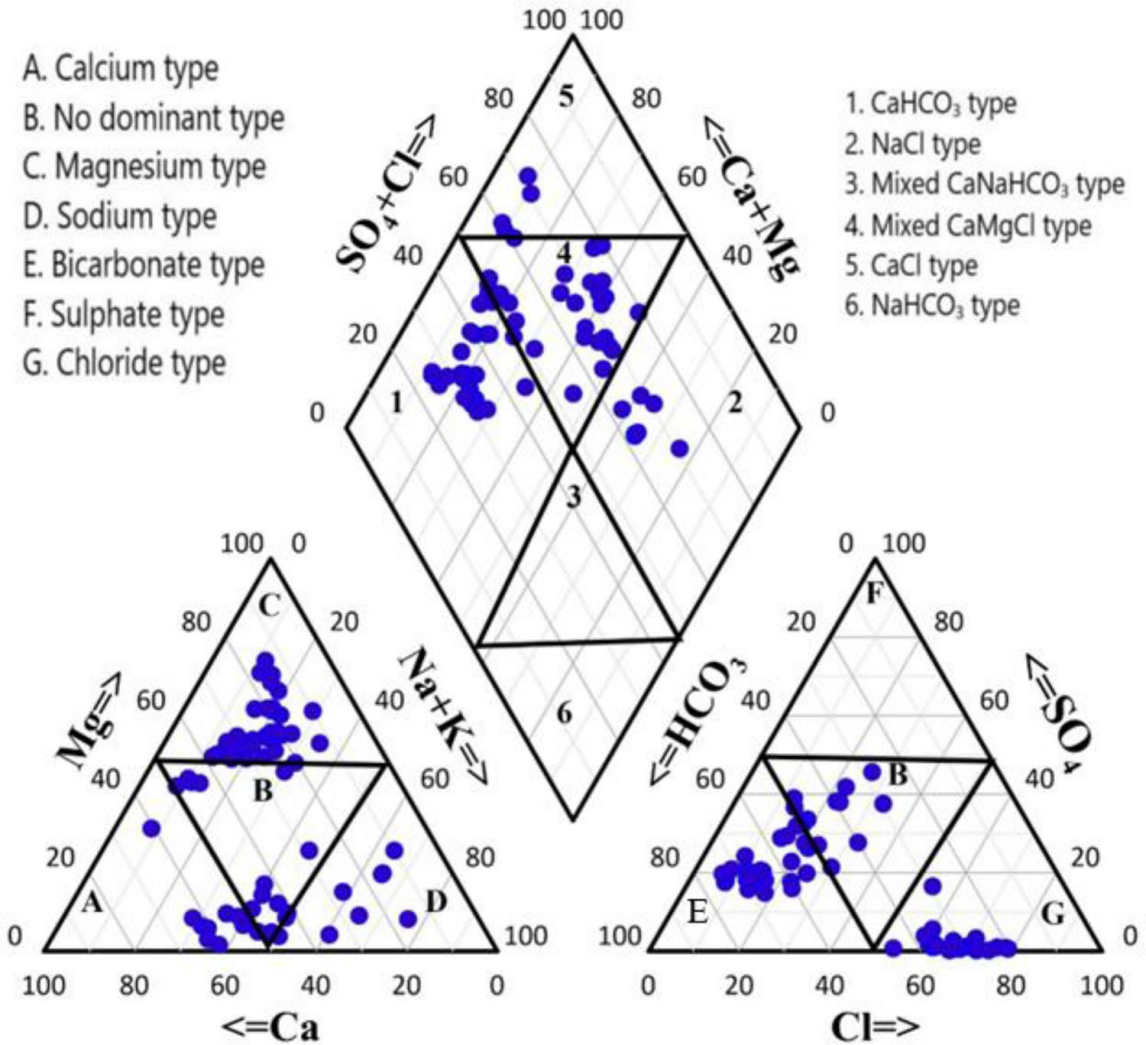
Piper diagram showing water type of the samples collected from the study area.

The overall groundwater condition inside a lithological structure is depicted by hydrochemical facies. They can be somewhat useful in figuring out how groundwater originates and flows^45^. The hydrochemistry of groundwater samples and their hydrochemical configuration are graphically depicted in Piper diagram (1944). To show the chemical differences between the groundwater samples, the samples were plotted on a Piper diagram as shown in (Fig. 3). In the present study 14 samples belong to CaHCO_3_ type, 5 samples belong to NaCl type, 20 samples belong to Mixed CaMgCl type, and 3 samples belong to CaCl type. It can be accredited due to weathering and recharge mechanism, evaporation processes, and reverse ion exchange^10, 22, 45^.

### Controlling mechanism of groundwater chemistry

**Figure 4:**
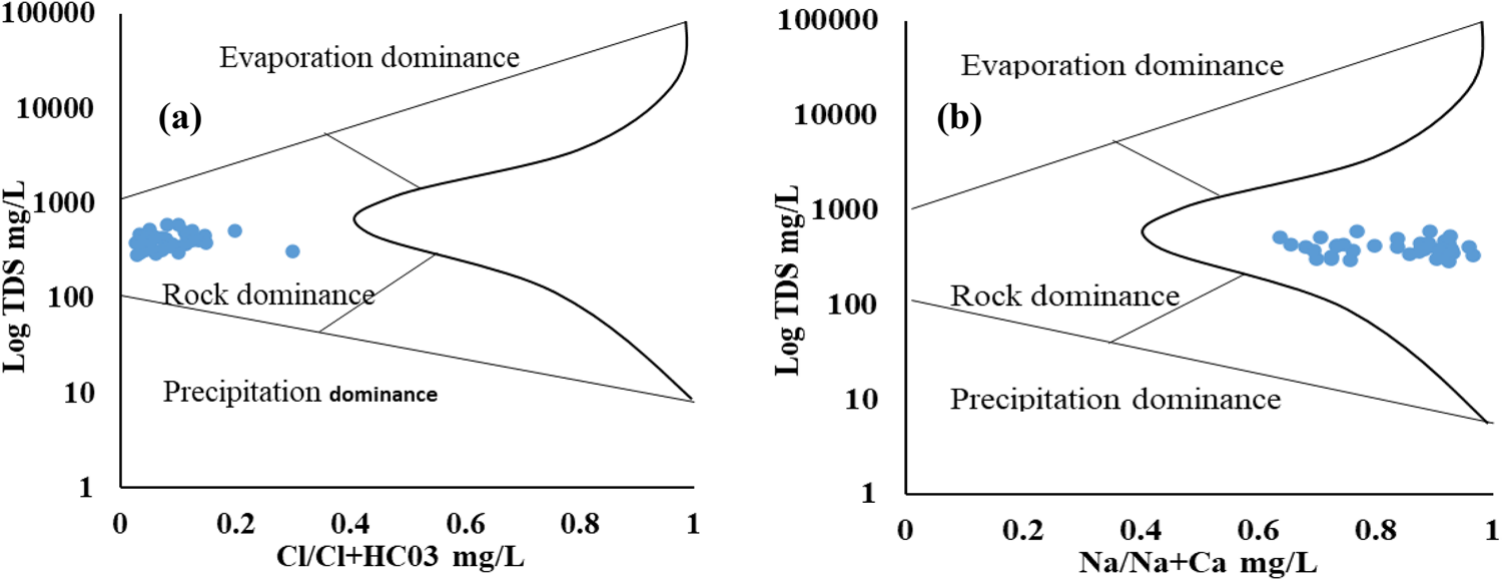
Gibbs diagram shows mechanism controlling groundwater chemistry (**a**) log TDS vs Na^+^/Na^+^  + Ca^2+^ mg/L, and (**b**) log TDS vs Cl^–^/Cl^–^ + HCO_3_^–^ mg/L.

Gibbs^46^, plots was used to figure out controlling mechanism of groundwater chemistry of the study area. Therefore, two plots were made, one for cations (Na^+^  + K^+^)/ (Na^+^  + K^+^  + Ca^2+^) as a function of total dissolved solids (TDS) (Fig. 4a), and the other for anions (Cl^–^/ (Cl^–^ + HCO_3_^–^) as a function of TDS (Fig. 4b). As demonstrated in (Fig. 4), all of the water samples in the research region are dominated by rock dominance. It is apparent from the outcome that rock weathering, and rock–water interaction are the key contributors to release As in the groundwater of the studied area.

The As release may result from edge reaction of rock-water interaction. In the current investigation, we identify the possible synthesis of CO_3_^-^containing minerals and their participation in As discharge under favorable alkaline condition^47^. The process by which the groundwater mixed dissolvable salts and minerals promoted the weathering of the parent rock. In addition, a long rock–water contact residence time also allows mineral dissolution. Therefore, aquifer lithology and groundwater bedrock mineralogy have been highlighting as geochemical processes of importance in the study area. The current findings were compared with^3^ and^1^ who also observed high As levels which was controlled by rock dominance zone.

### Saturation indices

**Figure 5:**
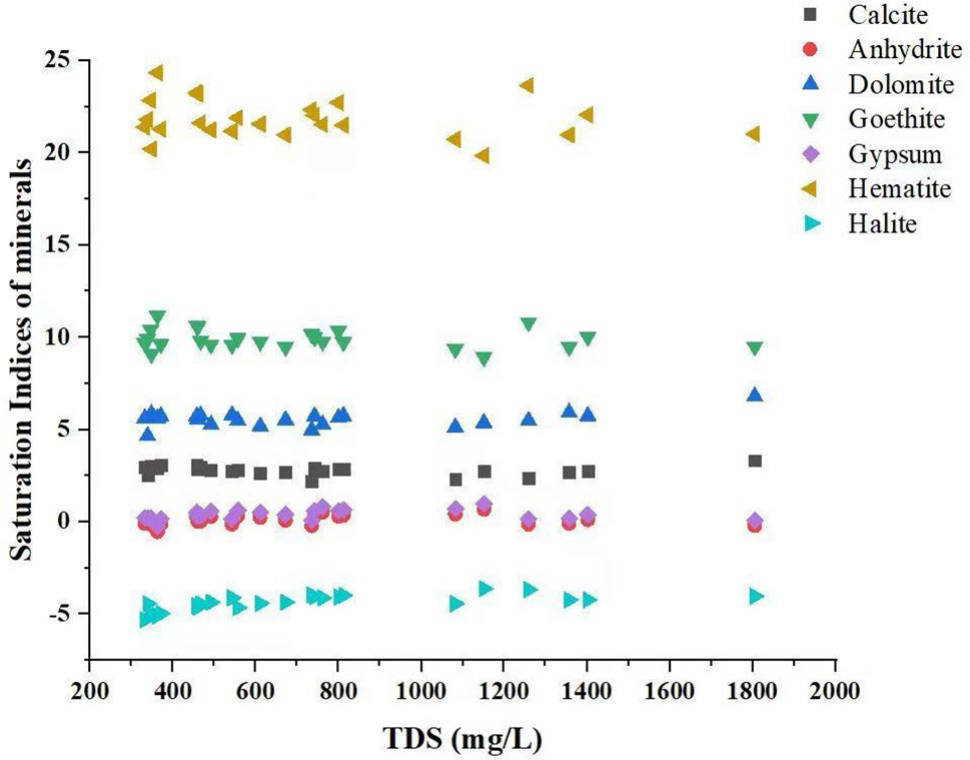
Saturation indices of Calcite, Anhydrite, Dolomite, Goethite, Gypsum, Hematite and Halite versus TDS in groundwater samples collected from the study area.

The presence of various solutes originating from the atmosphere or from rock weathering and erosion influences natural groundwater quality. Mineral species dissolve due to the contact between rock and water. Groundwater is saturated with a single mineral species under equilibrium conditions; however, it may continue to dissolve more minerals, which may eventually precipitate, and the water becomes too saturated with that mineral. The saturation index (SI) can be used to determine chemical equilibrium for a specific mineral species. Subsurface minerals are estimated using saturation data. As shown in (Fig. 5) the results of mineral phases in the groundwater resources of the study area, such minerals include Calcite, anhydrite, dolomite, goethite, gypsum, hematite, and halite minerals. The SI values of such minerals were observed in the range of, calcite (2.3187–3.325), anhydrite (− 0.0991–0.4174), dolomite (4.6648–6.804), geothite (9.095–11.1613), gypsum (− 0.2451–0.961), hematite (19.8496–24.3333), and halite (− 3.9558–− 5.0923), respectively. From the results its apparent that the groundwater sources of the study area were saturated for calcite, hematite, goethite, and dolomite, while gypsum and anhydrite were found in equilibrium surface and halite was found in under-saturated condition. The result of the present research was compared with the study of^1^ and^3^, who has observed high concentration of As with the saturation of calcite, hematite, goethite, and dolomite minerals.

### Principal component analysis (PCA) and Pearson correlation

Principal component analysis (PCA) technique was applied to assess all geochemical processes occurring in the study area. Table 2 displays the PCA results for 42 groundwater samples. The PCA was measured through the varimax rotation reduction dimension method. The R^2^ values were taken from the model summary, then the R^2^ of individual factors F1, F2, F3, F4, and F5 were calculated by removing one component and leaving all the other components as independent values. After the R^2^ calculation, the R^2^ difference was estimated by subtracting the R^2^ value of each element from the overall R^2^ values. The percentage contribution was calculated by summing the R^2^ differences following to^48^.

**Table 2:** Principal component analysis of groundwater variables in Bahawalpur district, Punjab Province, Pakistan.

Parameters	F1	F2	F3	F4	F5
pH	− 0.52	− 0.48	− 0.49	− 0.27	− 0.19
EC	**0.95**	− 0.21	− 0.16	− 0.10	0.10
Turbidity	0.14	**0.83**	− 0.17	− 0.28	− 0.09
TDS	**0.95**	− 0.21	− 0.16	− 0.10	0.10
Depth	0.30	− 0.20	**0.68**	0.26	− 0.38
Na^+^	**0.81**	− 0.39	− 0.29	− 0.21	0.04
K^+^	**0.76**	− 0.18	0.24	− 0.34	− 0.23
Cl^–^	0.37	**0.58**	0.29	0.31	− 0.02
HCO_3_^–^	**0.90**	− 0.15	0.02	− 0.05	0.13
SO_4_^2–^	**0.59**	**0.51**	− 0.21	0.34	0.18
Mg^2+^	**0.83**	− 0.07	0.05	0.39	0.22
Ca^2+^	0.29	**0.68**	0.17	− 0.49	− 0.20
NO_3_^–^	− 0.27	0.31	− 0.37	− 0.04	**0.64**
Fe^2+^	− 0.20	− 0.28	0.43	− 0.04	0.60
F^–^	− 0.17	− 0.08	− 0.38	**0.58**	− 0.26
As	− 0.39	− 0.11	**0.62**	− 0.17	0.32
Eigen value	5.76	2.51	1.93	1.37	1.34
Variability (%)	35.99	15.72	12.07	8.55	8.37
Cumulative %	35.99	51.71	63.78	72.33	80.69

Significant values are in bold.

**Figure 6:**
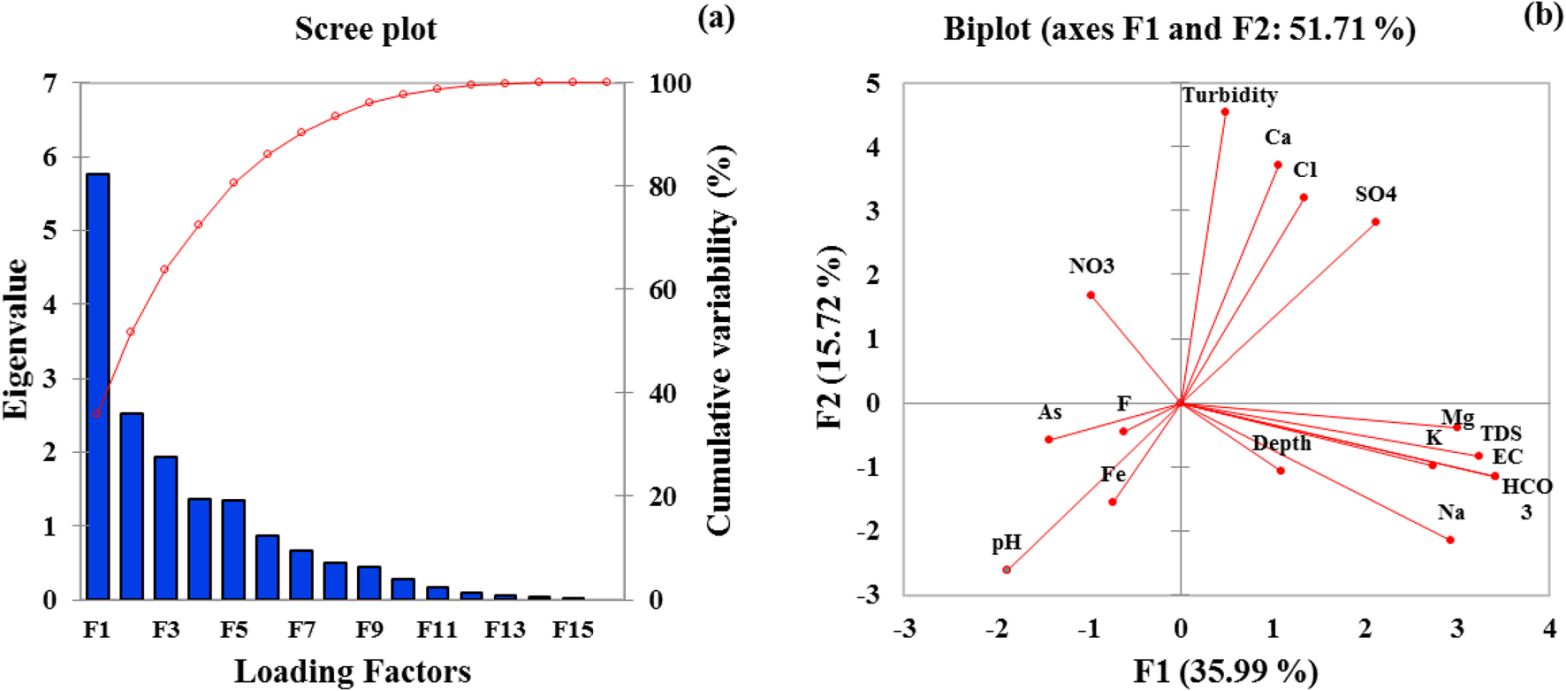
(**a**) Loading factors of the PCA–MLR results. (**b**) Relationship between the first two loading factors (F1 and F2) after Varimax rotation.

This analysis demonstrates five rotating components namely F1, F2, F3, F4, and F5, elucidating total variability (80.69 percent). In comparison, the total the loading factors were achieved to be 15 as indicated in Fig. 6a. Each factor’s variability: F1 described 35.99%, F2 described 15.72%, F3 described 12.07%, F4 described 8.55%, and F5 described 8.37%, respectively, with eigenvalues of 5.76, 2.51, 1.93, 1.37, and 1.34, respectively. However, the first two components F1 and F2 were built in a biplot and shows 51.71% variability as shown in Fig. 6b. The pollution index (PI) was calculated for overall groundwater parameters.

F1 shows variability of 35.99%, with eigenvalue of 5.76, and shows strong loading for: EC, TDS, Na^+^, K^+^, HCO_3_^–^, SO_4_^2–^ and Mg^2+^, respectively, with coefficient (R^2^) values: 0.95, 0.95, 0.81, 0.76, 0.90, 0.59, and 0.83, respectively. The source of Na^+^ and HCO_3_^–^ in groundwater resources is due to weathering and dissolution of carbonate and albite minerals. Similarly, TDS and EC originate from the influence of erosion of rocks having sulfide strata^49^. Furthermore, high TDS levels in the groundwater demonstrated ion dissolution, which could be due to the steady loss of salts and minerals over time^50^. The source of Mg^2+^ and K^+^ are carbonate weathering and K^+^ bearing rocks especially clay minerals^34^. Thus, F1 reflects for geogenic source of contamination arising in the study area.

F2 was counted with variance of 15.72% having an eigen value of 2.51 and shows strong loading for turbidity, Cl^–^, SO_4_^2–^ and Ca^2+^ with (R^2^) values of 0.83, 0.58, 0.51, and 0.68 respectively. The high turbidity in groundwater resources is due to poorly built wells^51^. Moreover, weathering processes, industrial effluents, surface runoff also results in high turbidity in groundwater resources^49^. The source of Cl^-^ in groundwater resources depend on many factors such as weathering, leaching of sedimentary rocks, and anthropogenic sources such as animal manure, fertilizers, and landfill leachates^33^. The source of SO_4_^2–^ in groundwater resources is due to mineral dissolution as well as mining activities and fertilizers. The most common contributor of SO_4_^2–^ is gypsum^52^. The source of Ca^2+^ in groundwater is rock-water interaction and mineralization which may contribute to high Ca^2+^ concentrations in groundwater^53^. F2 shows that the contamination of groundwater resources of the study area is of mixed type which may include both geogenic and anthropogenic sources occurring in the study area.

Similarly, F3 show 12.07% variability with an eigenvalue of 1.93 and shows strong loading for depth and As with (R^2^) values of 0.68 and 0.62 respectively. The correlation among depth and As shows that there is a strong contribution of the microbial activity in the mobilization of As. Arsenite mobilizes with increasing of borehole depth, which is also reported by^54^. Moreover, the correlation between the depth of wells and As levels can vary depending on the specific geological and hydrological conditions of the area. In some cases, deeper wells may have lower levels of As because they tap into deeper, uncontaminated groundwater resources. However, in other instances, deeper wells may encounter As rich aquifers or geological formations, resulting in higher As levels. It is important to note that the correlation between well depth and As is not universally consistent and can be influenced by various factors such as geological composition, hydrological dynamics, and local contamination sources^54^, thus F3 shows geogenic source of contaminant in the groundwater resources of the study area. F4 was counted with variance of 8.55% having an eigenvalue of 1.37 and shows moderate positive loading for fluoride (F^–^). The source of F^-^ in groundwater resources is due to fluorite bearing minerals which may result in the elevation of higher F^–^ in groundwater resources. Furthermore, there are many factors which may result in elevation of F^–^ in groundwater, such as cation exchange, evaporation, elevated concentrations of HCO_3_^–^ and Na^+^, and base ionic exchange mechanism^52^. F4 shows geogenic source of contaminant concerning HCO_3_^–^ and Na^+^ in the study area aquifers. Likely F5 show variability of 8.37% with eigenvalue of 1.34 and shows strong loading for NO_3_^–^ with (R^2^) value of 0.64. The source of high NO_3_^–^ levels in groundwater resources has been attributed to the overuse of pesticides/fertilizers and the use of wastewater for irrigation^31^. F5 shows anthropogenic source of contamination in groundwater resources of the study area. Thus, from the PCA results its concluded that the groundwater resources of the study area were contaminated due to anthropogenic and geogenic activities arising in the study area. Such sources of contamination include wastewater recharge, industrial effluents, agricultural practices, mining actions, weathering of rocks and rock-water interaction.

Table 3 shows moderate and strong positive and negative correlation among groundwater variables. The significant correlation values were observed among pH-EC (r = 0.540), pH-TDS (r = 0.540), pH-Cl^–^ (r = 0.557), pH-HCO_3_^–^ (r = 0.512), pH-SO_4_^2–^ (r = 0.608), pH-Mg^2+^ (r = 0.674), EC-TDS (r = 1.000), EC-Na^+^ (r = 0.896), EC-K^+^ (r = 0.579), EC-HCO_3_^–^ (r = 0.935), EC-SO_4_^2–^ (r = 0.757), EC-Mg^2+^ (r = 0.910), Turbidity-Ca^2+^ (r = 0.650), TDS-Na^+^ (r = 0.896), TDS-K^+^ (0.579), TDS-HCO_3_^–^ (r = 0.935), TDS-SO_4_^2–^ (r = 0.757), TDS-Mg^2+^ (0.910), depth-K^+^ (r = 0.525), depth-NO_3_^–^ (r = − 0.546). Na^+^-HCO_3_^–^ (r = 0.818), Na^+^-SO_4_^2–^ (r = 0.673), Na^+^-Mg^2+^ (r = 0.779), K^+^-HCO_3_^–^ (r = 0.505), K^+^-Mg^2+^ (r = 0.520), Cl^–^SO_4_^2–^ (r = 0.630), HCO_3_^–^Mg^2+^ (r = 0.847), SO_4_^2–^Mg^2+^ (r = 0.737), Fe^2+^-As (r = 0.661).

**Table 3:** Correlation analysis of selected parameters in groundwater sources of Bahawalpur district, Punjab Province, Pakistan.

	pH	EC	Turbidity	TDS	Depth	Na^+^	K^+^	Cl^–^	HCO_3_^–^	SO_4_^2–^	Mg^2+^	Ca^2+^	NO_3_^–^	Fe^2+^	F^–^	As
pH	1.00															
EC	0.540**	1.00														
Turbidity	− 0.11	0.01	1.00													
TDS	0.540**	1.000**	0.01	1.00												
Depth	− 0.30	0.20	− 0.32	0.20	1.00											
Na^+^	− 0.35	**0**.896**	− 0.16	0.896**	0.20	1.00										
K^+^	− 0.39	0.579**	− 0.10	0.579**	0.525**	0.494*	1.00									
Cl^–^	0.557**	0.39	0.13	0.39	0.33	0.29	0.26	1.00								
HCO_3_^–^	0.512**	**0**.935**	0.12	0.935**	0.16	0.818**	0.505*	0.34	1.00							
SO_4_^2–^	0.608**	0.757**	0.21	0.757**	0.05	0.673**	0.17	0.630**	0.329	1.00						
Mg^2+^	0.674**	0.910**	0.04	0.910**	0.28	0.779**	0.520**	0.428	0.847**	0.737**	1.00					
Ca^2+^	− 0.26	0.19	0.650**	0.19	0.04	− 0.07	0.21	0.23	0.28	0.30	0.09	1.00				
NO_3_^-^	0.00	− 0.07	0.27	− 0.07	− 0.546**	− 0.16	− 0.39	0.08	− 0.12	− 0.01	− 0.08	− 0.13	1.00			
Fe^2+^	− 0.02	− 0.20	− 0.33	− 0.20	0.10	− 0.23	− 0.07	0.10	− 0.19	− 0.16	− 0.20	− 0.05	− 0.17	1.00		
F^–^	0.17	− 0.23	0.00	− 0.23	0.22	− 0.11	− 0.02	− 0.05	− 0.12	− 0.08	− 0.13	− 0.22	− 0.428	− 0.06	1.00	
As	− 0.02	− 0.31	− 0.18	− 0.31	− 0.05	− 0.410*	− 0.12	0.07	− 0.37	− 0.30	− 0.32	0.05	0.08	0.661**	− 0.20	1.00

*Correlation is significant at the 0.05 level (2-tailed).

**Correlation is significant at the 0.01 level (2-tailed).

Significant values are in bold.

The result shows the moderate correlation among pH and other physicochemical parameters suggest that pH has a vital role in the saturation of physicochemical parameters in the aquifers of the study area^3, 55^. The correlation among cations and anions suggest that they are originate from the same source in groundwater, such as ion exchange processes and weathering of rocks^56^.

### Human health risk assessment

Table 4 shows the chronic daily intake (CDI), adverse non-carcinogenic (HQ), and carcinogenic risk (CR) from As exposure in the research area. The CDI value for children in the study area varied between (1.44E–06 to 1.25E–03) with an average value of (4.16E–04), similarly for adults the CDI value varied between (1.83E–07 to 1.59E–04) having an average value of (5.28E–05). The HQ value for children in the study area ranges between (4.79E–03 to 4.18E + 00) with mean value of (1.39E + 00), while for adults the HQ values 6.09E–04 to 5.30E-01 with an average value of (1.76E–01). The CR value for children in the study area ranges between (2.16E–06 to 1.88E–03) with an average value of (6.24E–04), similarly for an adult the CR value varied between (2.74E–07 to 2.39E–04) with mean value of (7.93E–05). These findings demonstrated that drinking arsenic-contaminated water poses a significant health risk to most inhabitants in the research area. As a result, areas exposed to arsenic should take considerable measures to protect inhabitants from arsenic exposure. The HQ value of As in groundwater samples was higher than the permitted limit for children living in the study area.

**Table 4:** Describe non-carcinogenic and carcinogenic risk of arsenic through oral ingestion of groundwater.

		Min	Max	Median	Mean
CDI	Children	1.44E–06	1.25E–03	4.20E–04	4.16E–04
	Adults	1.83E–07	1.59E–04	5.33E–05	5.28E–05
Non-carcinogenic risk	Children	4.79E–03	4.18E + 00	1.40E + 00	1.39E + 00
	Adults	6.09E–04	5.30E–01	1.78E–01	1.76E–01
Carcinogenic risk	Children	2.16E–06	1.88E–03	6.29E–04	6.24E–04
	Adults	2.74E–07	2.39E–04	7.99E–05	7.93E–05

### Suitability assessment of groundwater for drinking purposes

The water quality index (WQI) is a fundamental approach for determining the overall drinking water quality of ground and surface water. WQI has been widely used to quantify the total influence of hydrochemical factors on drinking water quality. The WHO-recommended WQI designates groundwater quality based on significant standards used for drinking purposes. Based on WQI result the quality of groundwater sources for drinking purpose is categorized in (Table 5), in which 11 samples belong to very poor category, 13 samples belong to poor category, 16 samples belong to good category and 2 samples belong to excellent category of groundwater resources samples of the study area for drinking purposes. From the result its concluded that most of the samples belong poor category and posing an adverse health effect on the population of the study area.

**Table 5:** Classification of groundwater water quality for drinking purposes.

WQI	Water type	No. of samples
(< 50)	Excellent	2
(> 50)	Good	16
(> 100)	Poor	13
(> 200)	Very poor	11

## Conclusion

The presence of elevated concentrations of arsenic in drinking water sources may make it unfit for human consumption and has a negative impact on human health. The research revealed that 27 of the 37 samples examined were above the WHO-recommended safe drinking limit of 10 µg/L. The As levels in the drinking water samples from tube-wells ranged from 0.12 to 104 µg/L, with an average of 34.7 µg/L. The source of As in groundwater of the study area is rock-water interaction, agricultural pesticides, weathering of rocks, and wastewater recharge. Statistical analysis result show that the groundwater cations values were in decreasing order such as: Na^+^ > Mg^2+^ > Ca^2+^ > K^+^, while anions were: HCO_3_^–^ > SO_4_^2–^ > Cl^–^ > NO_3_^–^. Hydrochemical facies result depict that the groundwater samples of the study area 14 samples belong to CaHCO_3_ type, 5 samples belong to NaCl type, 20 samples belong to Mixed CaMgCl type, and 3 samples belong to CaCl_2_ type. It can be accredited due to weathering and recharge mechanism, evaporation processes, and reverse ion exchange. Gibbs-diagram shows that rock-water interaction controls the geochemistry of groundwater sources of the study area. Saturation indices results indicated the saturation of calcite, dolomite, gypsum, geothite, and hematite mineral due their positive SI values. The principal component analysis (PCA) results possess a total variability of 80.69% suggesting that anthropogenic and geogenic contributing source of contaminant. The results of the Exposure-health-risk-assessment model for measuring As reveal significant potential non-carcinogenic risk (HQ) exceeding the threshold level of (value > 1) for children in the study area. Water quality assessment results shows that 24 samples were un-suitable for drinking purposes. To prevent further groundwater degradation and human suffering, it is critical to focus on monitoring and careful management of existing groundwater resources. According to our findings, water management authorities should establish a comprehensive groundwater system monitoring network. This will allow for real-time monitoring of groundwater quality and quantity, as well as the development of preventative actions. Local governments should advocate for more active and severe measures to create safe drinking water wells. Effective initiatives should also be taken to promote public understanding of the importance of using groundwater responsibly and safely.

### Supplementary Information

Supplementary Table S1.

## Data Availability

Data will be provided upon request to the corresponding author.
