# Supplementary material for: Integrated approach to hydrogeochemical appraisal of groundwater quality concerning arsenic contamination and its suitability analysis for drinking purposes using water quality index
Source: Sci Rep. 2023 Nov 22;13:20455. doi: 10.1038/s41598-023-40105-9 (PMC10665467; doi:10.1038/s41598-023-40105-9)
Supplement: Supplementary file 1 — Supplementary Table S1. [file 41598_2023_40105_MOESM1_ESM.docx]

**Table S1 -** Water quality parameters and methods used for analysis.

| **Sr. #** | **Parameters** | **Analytical Methods** |
| --- | --- | --- |
| 1 | Alkalinity (mg/L as CaCO_3_) | 2320, Standard method (2005) |
| 2 | Arsenic (μg/L) | AAS Vario 6, Analytik Jena AG (3111B APHA) |
| 3 | Bicarbonate (mg/L) | 2320, Standard method (2005) |
| 4 | Calcium (mg/L) | 3500-Ca-D, Standard Method (2005) |
| 5 | Carbonate (mg/L) | 2320, Standard method (2005) |
| 6 | Chloride (mg/L) | Titration (Silver Nitrate), Standard Method (2005) |
| 7 | Conductivity (μS/cm) | E.C meter, Hach-44600-00, USA |
| 10 | Magnesium (mg/L) | 2340-C, Standard Method (2005) |
| 11 | Nitrate as Nitrogen (mg/L) | Cd. Reduction (Hach-8171) by Spectrophotometer |
|  |  |  |
| 12 | pH | pH Meter, Hanna Instrument, Model 8519, Italy |
|  |  |  |
| 13 | Sodium (mg/L) | Flame photometer PFP7, UK |
| 14 | Sulfate (mg/L) | SulfaVer4 (Hach-8051) by Spectrophotometer |
|  |  |  |
| 15 | TDS (mg/L) | 2540C, Standard method (2005) |
| 16 | Turbidity (NTU) | Turbidity Meter, Lamotte, Model 2008, USA |
| 17 | Fluoride (mg/L) | 4500-FC.ion-Selective Electrode Method Standard (2005) |
| 18 | Iron (mg/L) | Ferro Ver method (HACH Cat. 21057-69) |
|  |  |  |
|  |  |  |
|  |  |  |
|  |  |  |
|  |  |  |
